# Rapid Shifts of Peak Flowering Phenology in 12 Species under the Effects of Extreme Climate Events in Macao

**DOI:** 10.1038/s41598-018-32209-4

**Published:** 2018-09-17

**Authors:** Jianhao Zhang, Qifei Yi, Fuwu Xing, Chunyan Tang, Lin Wang, Wen Ye, Ian Ian Ng, Tou I Chan, Hongfeng Chen, Dongming Liu

**Affiliations:** 1 South China Botanical Garden, The Chinese Academy of Sciences, Guangzhou, 510650 China; 2 University of Chinese Academy of Sciences, Beijing, 100049 China; 3 Department of Garden and Green Areas, Civil and Municipal Affairs Bureau of Macao Special Administrative Region, Macao, 999078 China

## Abstract

Plant phenology is sensitive to climate change; the timing of flowering has served as a visible indicator of plant phenology in numerous studies. The present study used phenological records from a manual monitoring program to characterize the flowering phenology of 12 species in Guia Hill, Macao. The mean peak flowering dates (PFDs) of these species ranged from March to September, 41.7% of which occurred in May. The earliest or latest PFDs of nine species occurred in 2013, a year with extremely heavy rain events in early spring. In addition, we found that, in the 5-year period, the monthly mean temperature or monthly precipitation in two periods, specifically 1) during November to December of the previous year and 2) during 0–2 months before the PFDs of each species, were significantly correlated with the PFD of eight species. The result showed that, even though complex species-specific responses to the characteristics of climate widely exist, most species in the present study responded to shifts in climate shifts in these two periods. In addition, some species were extraordinarily sensitive to extreme climate events. Precipitation was more effective in altering flowering date than temperature, especially among the late-flowering species in Guia Hill, Macao.

## Introduction

Phenology, the timing of recurring events in the lives of plants and animals and their relationships with climate^1^, has received an increasing amount of attention throughout the world in the light of climate change. Thus, phenological monitoring has become a very valuable tool used to assess the effects of climate change^2^. Both internal controls and environmental factors determine the phenophase of a plant. An array of abiotic factors may affect plant phenology. Temperature^3,4^ and photoperiod^5,6^ are widely accepted as the proximal abiotic stimuli for phenological changes in temperate regions, such as England^7^, Germany^8^, and North China^9^. However, precipitation has been also considered to be an important factor that affects phenology in tropical and subtropical areas^10–12^, arid areas^13,14^, and the Mediterranean region^15,16^.

Flowering events is easily observable, especially in southern subtropical forests where fully expanded leaves remain on many plants almost all year round, and senescence is not as distinct as happens in temperate areas. Meanwhile, flowering is a crucial phenophase of the reproductive process of individuals that affects their success and the long-term persistence of populations^17,18^. Changes in the flowering period affect other related processes such as pollination, seed ripening, dispersal, and germination^19^; these changes also affect the activity of many relevant animals^20^. Therefore, the variability in flowering time among species has a potential effect on competitive interaction and even on niche differentiation^21^. Based on the above, we focused on flowering phenology as an important indicator of climate change in the present study. A number of studies have revealed that for most species the onset of spring phenological events has advanced to earlier in the season over the past decades^8,22^ as plants respond to global warming; however, some species have exhibited little change in the timing of flowering or flowering has occurred even later in other species^7,23^. The sensitivities of different functional traits to climate change of different species also varied. For example, early-flowering species reacted more strongly and sensitively to climate change than late-flowering species^24–26^. Plants with different pollination types^27–29^ and different phylogenetic relationships^17,30^ use a variety of mechanisms to adapt to climate change.

In contrast with the gradual warming that is occurring along with climate change, extreme climate events are not necessarily a part of long-term climatic trends, such as heavy rainfall events, heat waves, or abnormal drought within several days or weeks; relatively few previous reports have addressed the effects of such events on phenology^31^. However, Both *et al*. believed short-term extreme events had a more powerful effect on disturbing the synchronization of activities and phenology among organisms than gradual warming^32^. In Kansas, the duration of flowering was compressed by a sudden rainfall event after a long period of drought^33^. Climate change induced by the North Atlantic Oscillation had a strong correlation with flowering phenophases in Germany^8^, Latvia, and Lithuania^34^. The timing of flowering and the number of flowers produced by *Styrax officinalis* were related to sudden shifts in previous rainfall events^35^. The occurrence of different types of extreme events and their return intervals are hard to predict^36^. Therefore, the present paper will focus on the effects of extreme events on phenology along with the effects of climate change.

In the spring of 2013, Southern China experienced several weeks of heavy rainfall with abnormally high precipitation in Macao from March to June^37^. During that time, Guia Hill received 1118.62 mm of precipitation, 159.22 mm more than the annual average during this time period from 1981 to 2010. In addition, the strong El Niño event in 2015 caused it to rain more frequently than average in southern China in the first half of 2016. The amount of the precipitation received at Guia Hill in January 2016 reached 250.13 mm, which was almost 8 times the average (31.31 mm) from 1981 to 2010 during the same period.

The present study was conducted as part of the Phenological Monitoring of Wild Plants in Macao project that started in October 2011, a project that continues currently. In the process of sorting raw phenological data, we found that some species (*Psychotria asiatica*, *Cinnamomum burmannii*, etc.) appeared to be related to the abnormal climate during the early spring of 2013. As a result, we hypothesized that periods of extreme precipitation would affect the flowering phenology in several species of plants by delaying or advancing the peak flowering dates of some species. and took a first step in analyzing the rapid shifts in peak flowering date during a 5-year period to fill in the gaps for phenological studies in Macao. Some climate-sensitive species will be filtered out in the present study.

## Materials and Methods

### Study Locations

Located along the southeast coast of China, the Macao Special Administrative Region borders on Guangdong Province and faces Hong Kong across the Pearl River Estuary to the east. The sea surrounds Macao on three sides. Data from the Macao Meteorological and Geophysical Bureau (http://www.smg.gov.mo) shows that Macao covers 30.4 km^2^ and has a subtropical oceanic monsoon climate with a mean annual temperature of 22.6 °C. In the 5-year study period, summertime temperatures (≥22 °C) in Guia Hill began in April and lasted for more than 7 months. With the exception of 2012, the coldest MMT was higher than 15 °C showing that the climatic of Macao was warm all year round with a long summer (from May to September). Relatively less precipitation fell from November to the next February, especially in January of 2014, no rainfall occurred. Precipitation in spring and summer was abundant, but variable, among the 5 years of the present study.

The vegetation type of Guia Hill is a subtropical monsoon evergreen broad-leaved forest. The present study used a 800 m^2^ sample area in a climax community that had naturally formed over a hundred years. This site lies in the woods close to Monte Fort (22°11′51.5″N, 113°32′47″E, 90 m a.s.l.) in Guia Hill Municipal Park, which is located in the center of Macao. The study site was divided equally into 32 plots of 5 m × 5 m. These plots were tagged in two groups as 1 *A* to 1 *P* and 2 *A to 2* *P* in sequence.

### Phenological and Meteorological Data

At the Phenological Monitoring of Wild Plants in Macao project site that includes the present study area, each adult individual (≥1 cm diameter at breast height, ≥1.5 m tall) within the plots was labeled with a serial number, such as *A1*, *A2… P27*, *P28*. Each plant was marked on a small aluminum tag; then we observed and periodically recorded the phenology of each individual (twice a month in spring and summer; once a month in autumn and winter). In addition, several species of common herbage, such as *Alocasia macrorrhiza* and *Lophatherum gracile*, were also included in the initial monitoring. Observations of each phenophase conformed to uniform criteria based on *Methods of phenology observation of China*^38^. We defined the midpoint in flowering for each individual as the point in time between the start and end dates of flowering as the peak flowering date (PFD) for each species^17^. Studies of reproductive phenology have often employed this method to reduce potential bias caused by interpreting the initial flowering dates related to different sampling frequencies or population sizes^24,25,39,40^.

From January 2012 to April 2017, 64-months of continuous phenological observational data were recorded (some late-flowering species bloomed into the next year, so four months in 2017 were included). Because a few individuals died or disappeared while others grew large enough to be labeled during the 5-year study period, and not every individual was observed to bloom every year, the total number of flowering individuals varied over year. We eliminated any species that were represented by fewer than 5 years flowering records. The number of individuals in Table 1 is the total number of all observed individuals existing in our sample area, but not each of them was observed to bloom every year. The PFD of any one species is the mean value of all PFDs for all flowering individuals.

**Table 1:** Number of individuals, peak dates of flowering, and interannual standard deviation (SD) of 12 species in Guia Hill, Macao.

Species	Number of individuals	Peak flowering date					Mean	Interannual SD
		2012	2013	2014	2015	2016		
*Psychotria asiatica*	197	150.35	137.14	155.41	152.45	144.84	148.04	7.21
*Sterculia lanceolata*	88	136.07	120.38	135.45	121.11	129.84	128.57	7.55
*Cinnamomum burmannii*	66	93.09	72.42	90.49	101.63	99.50	91.43	11.56
*Mallotus paniculatus*	38	241.50	231.79	243.42	244.25	232.00	238.59	6.20
*Desmos chinensis*	18	207.37	149.83	168.00	187.10	192.75	181.01	22.42
*Lophatherum gracile*	17	261.55	240.00	270.29	278.00	255.25	261.02	14.57
*Syzygium jambos*	14	102.00	94.13	103.50	98.92	85.75	96.86	7.17
*Benkara scandens*	13	115.13	133.05	125.38	112.23	120.00	121.16	8.32
*Triadica cochinchinensis*	7	138.79	130.13	144.38	130.70	157.00	140.20	11.11
*Syzygium levinei*	5	212.00	247.50	211.00	223.50	230.00	224.80	14.99
*Breynia fruticosa*	4	187.50	149.25	165.00	207.25	121.00	166.00	33.43
*Litsea monopetala*	4	109.75	110.00	94.67	150.75	146.00	122.23	24.72

We obtained the meteorological data used here directly from the Macao Meteorological and Geophysical Bureau. This included data for daily mean temperature and daily precipitation from the Monte Fort station, a location that is less than 1 km from the sample area. As a result, the meteorological data nearly reflect the real hydrothermal conditions around the sample area. The monthly mean temperature (MMT) and monthly precipitation (MP) were then calculated.

**Figure 1:**
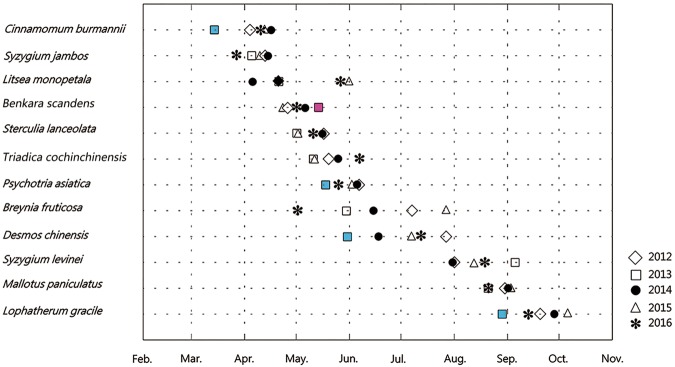
Shift in flowering phenology in 2012–2016 of 12 species in the present study. Each symbol represents a year; the date corresponding to each symbol in the horizontal coordinates is the peak flowering date of the year. The spacing between symbols reflects the concentration of peak flowering dates during the 5-year period. Significant advances and delays in flowering date are shown in blue and red, respectively.

**Figure 2:**
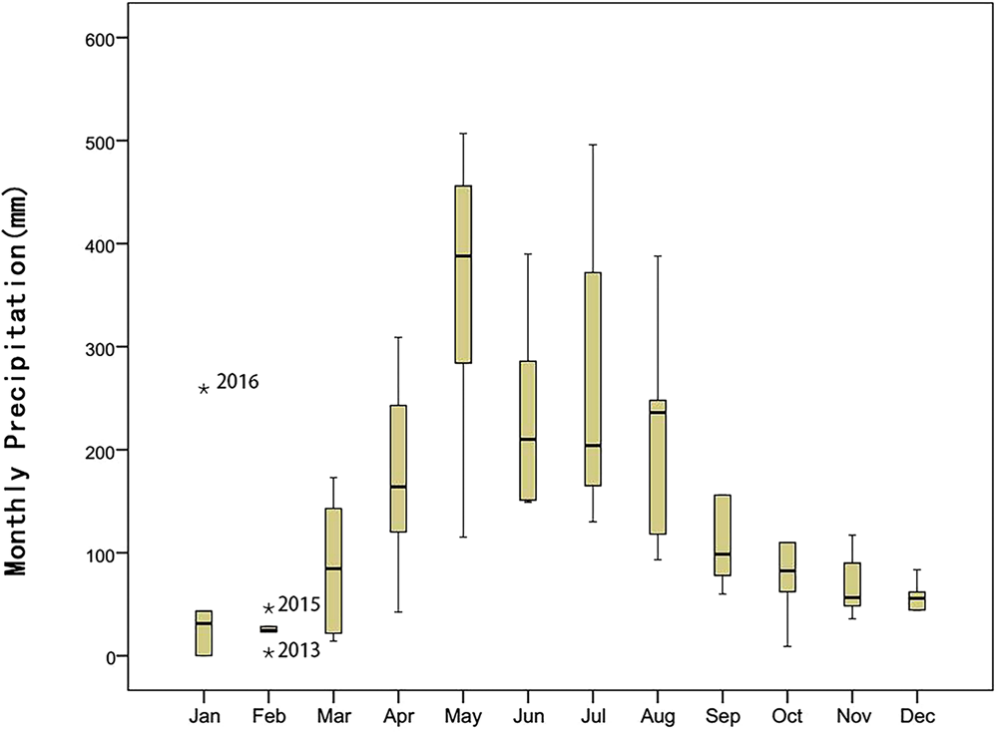
Boxplot of Monthly Precipitation (MP) of each month in 2012–2016. *Indicates the deviation was significant. The MP of January 2016 and February 2015 was significantly high. The MP of February 2013 was significantly low. Variation amplitudes of MP in March-August were obviously larger than other months.

**Figure 3:**
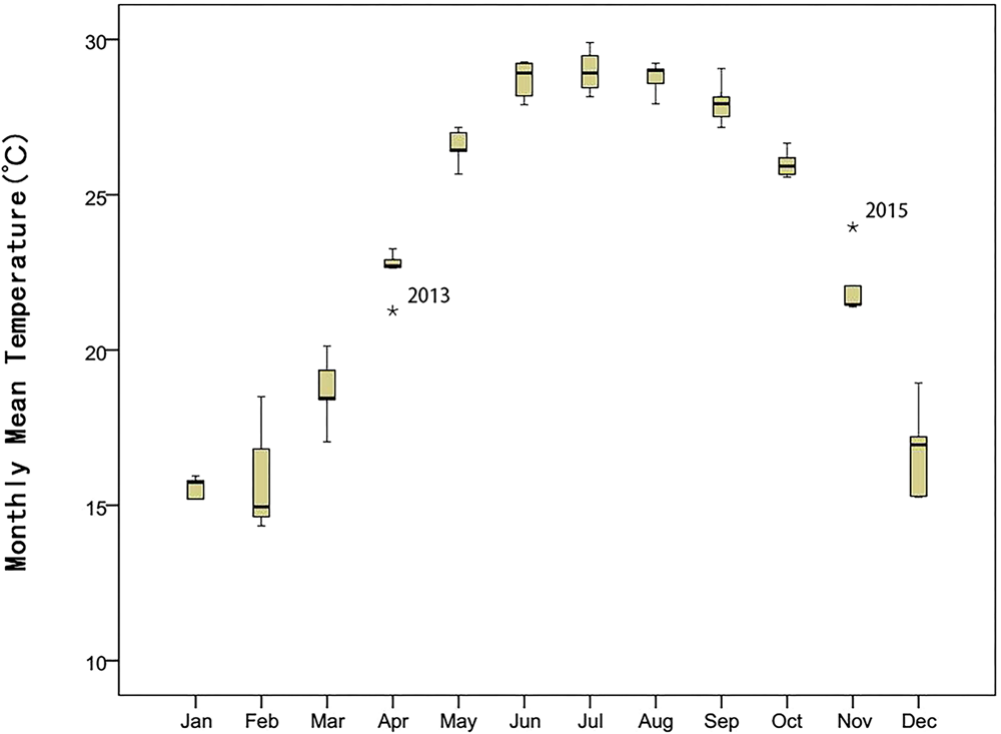
Boxplot of Monthly Mean Temperature (MMT) of each month in 2012–2016. *Indicates the deviation was significant. MMT in April 2013 was significantly low. MMT in November 2015 was significantly high. According to the correlation analysis of present study, the MMT in November 2015 mainly affected the peak flowering dates in 2016. The amplitudes of variation in MMT in February and December are larger than other months.

Thus, as fieldwork progressed, the annual PFDs of the 12 species were determined (Table 1 and Fig. 1). In this 5-year study period, nine species had extreme PFDs (the earliest and the latest FPD) in 2013 and three species had them in 2016. Only one species (*Litsea monopetala)* had extreme values in neither 2013 nor 2016. In order to determine the effects of abnormal climate on plant phenology in 2012 to 2016, the abnormal climate value was tested by Boxplot using SPSS software (Figs 2 and 3). Then, for these species whose extreme values were in 2013 or 2016, such as *Psychotria* *asiatica* for example, we divided all the FPDs for individual plants over the 5 years into two groups. PFDs in group 1 were from the extreme-value year (2013) while PFDs in group 2 were from other years. The significance of the difference between these two groups was tested by one-way ANOVA using SPSS. The same procedure was applied to the remaining ten species (not *L*. *monopetala*) and the results are presented in Tables 2 and 3.

**Table 2:** Peak flowering dates of nine species. The differences are between extreme values in 2013 and the values in other years.

Species	Advanced Days	Significance (P)
*Desmos chinensis*	38.9	0.004**
*Cinnamomum burmannii*	23.5	0.000**
*Lophatherum gracile*	26.4	0.006**
*Psychotria asiatica*	15.2	0.001**
*Triadica cochinchinensis*	12.6	0.42
*Sterculia lanceolata*	8.9	0.085
*Mallotus paniculatus*	8.5	0.247
*Benkara scandens*	−14.9	0.03^*^
*Syzygium levinei*	−29	0.007**

* and ** indicate a significant correlation at the 0.05 and 0.01 levels, respectively (2-tailed).

**Table 3:** Peak flowering dates of three species. The differences are between extreme values in 2016 and the values in other years.

Species	Advanced Days	Significance
*Breynia fruticosa*	54.9	0.247
*Syzygium jambos*	12.6	0.17
*Triadica cochinchinensis*	−21	0.159

* and ** indicate a significant correlation at the 0.05 and 0.01 levels, respectively (2-tailed).

Second, the timing of phenological events are strongly correlated with climate factors during a certain period before each event occurs^9,29,41^. We adopted Pearson’s correlation to test the significance of the correlation coefficients between each species’ PFDs and the MP and MMT from September of the previous year to its average PFD, respectively. The month whose MP or MMT correlated significantly (P value < 0.05) with PFDs was defined as the optimum period (OP). The relationship between FPDs and climate factors can usually be used as a measure of the sensitivity to different periods^9,42^.

## Results

**Figure 4:**
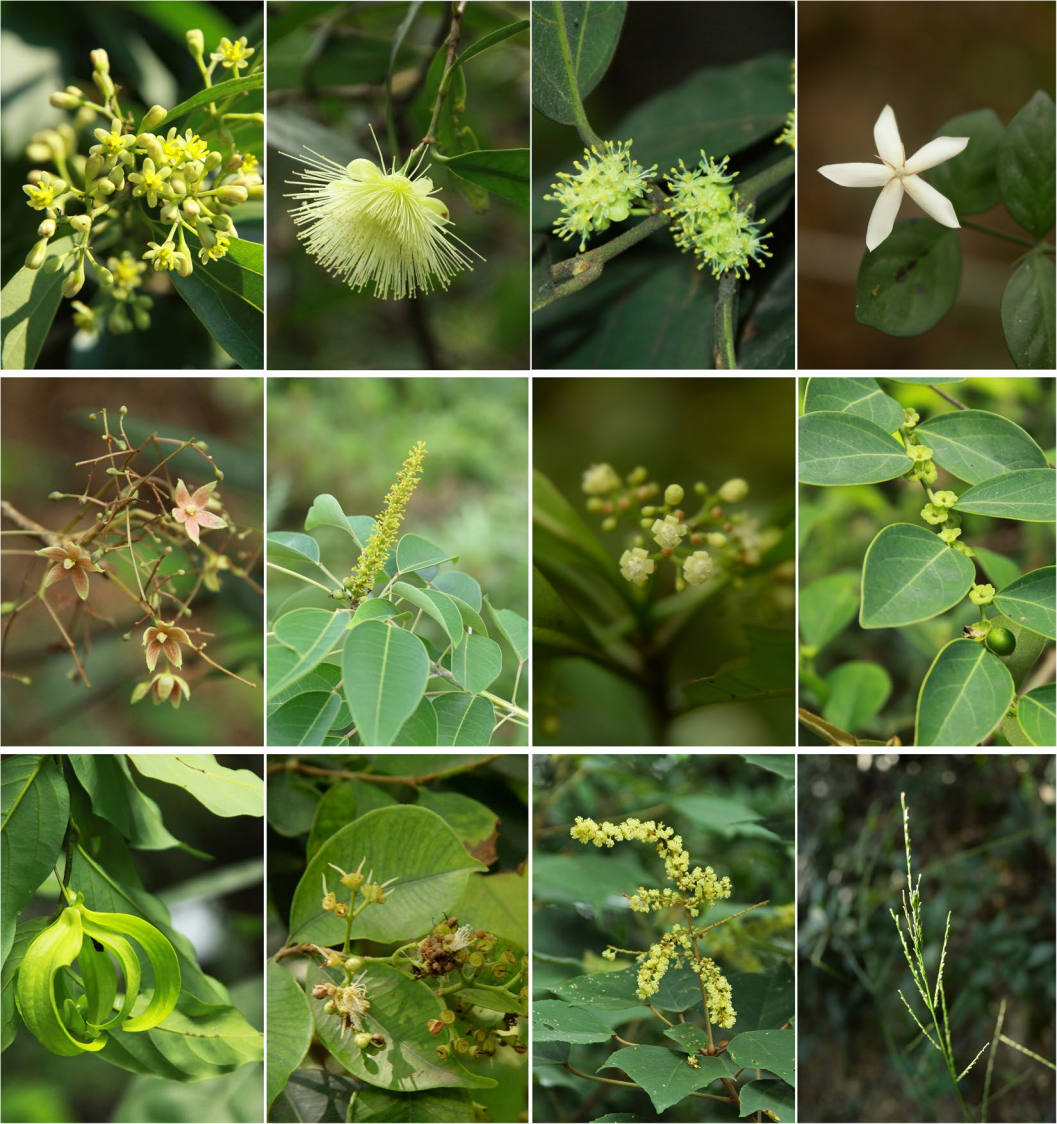
Twelve species in Guia Hill, Macao included in this study. From the top-left to bottom-right: *Cinnamomum burmannii, Syzygium jambos, Litsea monopetala, Benkara scandens, Sterculia lanceolata, Triadica cochinchinensis, Psychotria asiatica, Breynia fruticosa, Desmos chinensis, Syzygium levinei, Mallotus paniculatus, Lophatherum gracile*.

Most species bloomed in spring and summer, and the pictures of each species in the peak flowering period are presented in Fig. 4. The mean PFDs of this study ranged from 91.43 d (2 April) for *C*. *burmannii* to 261.02 d (19 September) for *L*. *gracile*. The mean PFDs of 5 of the 12 species analyzed here occurred in May. Considerable intraspecific variation was observed in the annual standard deviation (SD) of PFDs between species. The annual SD of *Breynia fruticosa* was the largest at 33.43 d, suggesting its PFD was the most scattered and varied widely among the 5 years. In contrast, the annual SD of *Mallotus paniculatus* (6.2 d) was the least, suggesting the PFDs of this species were varied little (Table 1 and Fig. 1).

During the 5-year study period, the earliest and latest PFDs occurred for seven and two species, respectively, in 2013. Among these, the PFDs of four species advanced significantly by more than 15 days when compared to the average PFDs of the other 4 years of analysis. Meanwhile, the PFDs of *Benkara scandens* and *Syzygium levinei* were significantly delayed by 14.87 days and 29 days, respectively. In 2016, two species had their earliest PFD and one species had its latest PFD, although these were insignificant changes. Therefore, the abnormal climate in 2013 might have had a more effective impact on PFDs than the climate in 2016, when reviewed from either the aspect of the number of species with extreme values or the significance of the differences (Tables 2 and 3).

The PFDs of 8 out of 12 species in Guia Hill were significantly correlated with monthly precipitation (Table 4) or monthly mean temperature (Table 5) in the OP within the previous half year and almost all of these OPs were distributed in two periods. Period-1 is November to December of the previous year, and period-2 is 0–2 months previous to their 5-year mean PFDs. Four species had a significantly negative correlation with MP in both periods-1 and -2, while *L*. *gracile* had a significantly negative correlation with MP in period-2 only. In particular, the significant correlation between *B*. *scandens* and MP was negative in February while being positive in March–May. *Breynia fruticosa* and *Desmos chinensis* had positive significant correlations with MMT in period-2 while *S*. *jambos* and *M*. *paniculatus* had negative significant correlations with MMT in period-1. The PFDs of *C*. *burmannii* were positively and significantly correlated with MP in period-2. Only one species, *Triadica cochinchinensis*, was significantly and negatively correlated with MMT in period-2.

**Table 4:** Pearson correlations between peak flowering dates and monthly precipitation from the last Sep.

	Last Sep.	Last Oct.	Last Nov.	Last Dec.	Last Fourth Quarter	Jan.	Feb.	Mar.	The First Quarter	Apr.	May	Mar.–May	Jun.	Jul.	Aug.	Sep.
*Cinnamomum burmannii*	0.192	0.447	−0.168	−0.627	0.245	0.485	0.975**	−0.274	0.261	−0.364						
*Syzygium jambos*	0.386	−0.809	−0.934*	0.108	−0.946*	−0.829	0.131	−0.826	−0.900*	0.005						
*Litsea monopetala*	−0.535	0.652	0.272	−0.145	0.554	0.597	0.577	0.089	0.468	−0.396	−0.515	−0.668				
*Benkara scandens*	0.073	−0.326	0.403	0.583	0.018	−0.224	−0.900*	0.601	0.063	0.113	0.713	0.981**				
*Sterculia lanceolata*	0.801	0.049	−0.137	−0.748	−0.055	0.13	0.215	−0.106	0.051	0.383	−0.208	−0.039				
*Triadica cochinchinensis*	0.54	0.654	0.592	−0.696	0.735	0.825	0.263	0.601	0.825	0.03	−0.264	0				
*Psychotria asiatica*	0.640	−0.296	−0.709	−0.358	−0.441	−0.193	0.767	−0.625	−0.350	−0.375	−0.018	−0.462				
*Breynia fruticosa*	−0.145	−0.516	−0.929*	0.121	−0.787	−0.652	0.445	−0.981**	−0.834	−0.154	−0.099	−0.564	−0.846	0.016		
*Desmos chinensis*	0.041	0.56	−0.123	−0.884*	0.205	0.443	0.703	−0.367	0.168	0.293	−0.894*	−0.849	−0.299	0.063		
*Syzygium levinei*	−0.698	0.178	0.612	0.597	0.38	0.118	−0.641	0.582	0.293	0.016	0.199	0.43	0.733	0.061	0.342	
*Mallotus paniculatus*	0.382	−0.539	−0.935*	−0.096	−0.733	−0.525	0.63	−0.874	−0.688	−0.319	0.045	−0.471	−0.982**	−0.205	−0.664	−0.622
*Lophatherum gracile*	0.367	−0.227	−0.709	−0.216	−0.387	−0.156	0.885	−0.666	−0.335	−0.568	−0.072	−0.632	−0.942*	−0.563	−0.35	−0.742

* and ** indicate a significant correlation at the 0.05 and 0.01 levels, respectively (2-tailed).

**Table 5:** Pearson correlations between peak flowering dates and mean monthly temperature from the last Sep.

	Last Sep.	Last Oct.	Last Nov.	Last Dec.	Last Fourth Quarter	Jan.	Feb.	Mar.	The First Quarter	Apr.	May	Mar –May	Jun.	Jul.	Aug.	Sep.
*Cinnamomum burmannii*	0.537	0.198	0.592	−0.355	0.238	−0.171	−0.649	−0.638	−0.615	0.854						
*Syzygium jambos*	−0.204	−0.455	−0.667	−0.83	−0.965**	−0.123	−0.024	0.381	0.087	0.19						
*Litsea monopetala*	0.868	0.609	0.591	0.158	0.639	0.102	0.024	−0.242	−0.044	0.229	0.259	0.041				
*Benkara scandens*	−0.59	0.064	−0.458	0.418	−0.034	0.43	0.422	0.36	0.472	−0.828	−0.941*	−0.667				
*Sterculia lanceolata*	−0.666	−0.665	0.255	−0.17	−0.173	−0.54	−0.86	−0.647	−0.835	0.687	0.424	0.023				
*Triadica cochinchinensis*	−0.332	0.023	0.741	0.361	0.621	−0.138	−0.828	−0.953*	−0.811	0.499	0.025	−0.61				
*Psychotria asiatica*	0.106	0.227	0.498	0.011	−0.506	−0.052	−0.604	−0.297	−0.439	0.726	0.748	0.563				
*Breynia fruticosa*	0.446	−0.167	−0.43	−0.8	−0.71	−0.146	0.192	0.46	0.221	0.266	0.68	0.982**	0.035	−0.247		
*Desmos chinensis*	0.234	−0.391	0.705	−0.085	0.229	−0.75	−0.644	−0.623	−0.773	0.917*	0.869	0.426	−0.116	0.076		
*Syzygium levinei*	0.192	0.431	−0.064	0.679	0.456	0.373	0.748	0.436	0.654	−0.832	−0.786	−0.515	−0.239	−0.504	−0.6	−0.68
*Mallotus paniculatus*	0.181	−0.156	−0.359	−0.963**	−0.747	−0.053	−0.253	0.098	−0.114	0.521	0.742	0.819	0.356	0.27	0.839	0.816
*Lophatherum gracile*	0.409	0.165	−0.052	−0.869	−0.408	0.088	−0.41	−0.176	−0.253	0.653	0.767	0.645	0.645	0.515	0.514	0.653

* and **indicate a significant correlation at the 0.05 and 0.01 levels, respectively (2-tailed).

## Discussion

The PFDs of two species (*L*. *monopetala* and *Sterculia lanceolata*) did not vary significantly in 2013 (or 2016) and lacked significant correlation with MP or MMT in the 5-year study period. This suggests that their PFDs are relatively insensitive to precipitation or temperature; perhaps a complex mechanism with genetic controls that is controlled by other environmental factors, such as photoperiod, etc. As for *P*. *asiatica* and *S*. *levinei*, although their correlations with MMT and MP were not significant, their earliest PFDs in 2013 advanced significantly when compared with those of other years. Not all climate changes affected these two species until temperature and precipitation shifted far enough to cause changes. In contrast, the PFDs of *M*. *panic*ulatus and *S*. *jambos* were negatively and significantly correlated with both MMT and MP, but their PFDs did not advance significantly in 2013 (or 2016). This implies their PFDs could be advanced by abundant rainfall and warm weather within a certain range, but could also be regulated and constrained by other biotic and abiotic factors; as a result, their PFDs were relatively stable under abnormal climatic conditions. The PFDs of *Breynia fruticosa* and *D*. *chinensis* were positively correlated with MMT in period-2 but were negatively correlated with MP in both periods-1 and -2. It has been reported that warming could expand the reproductive period of some species^43,44^; here, one cause may be that higher temperature prolonged the flowering of these species so the PFDs were delayed accordingly. The interannual SDs of *B*. *fruticosa* and *D*. *chinensis* lasted for more than 20 days, and the PFDs advanced by 54.9 days and 38.9 days in the earliest year, respectively, which showed the PFDs of two species varied rapidly and tracked the MP and MMT in OPs tightly. However, the deciduous tree *T*. *cochinchinensis* was the only species with a PFD that was negatively correlated with MMT in period-2, which means higher spring temperatures could cause this species to flower earlier, like other species originating from temperate regions^42,45^. The mean PFD of *C*. *burmannii* was in April and its PFDs were positively and significantly correlated with MP in February. This implies that the PFDs could be retarded by rainfall in period-2. Another piece of evidence is that PFDs of *C*. *burmannii* significantly advanced in 2013, with precipitation in February being extremely low. In particular, the PFD of *B*. *scandens* ranged from April to May and was significantly correlated with precipitation in February while this correlation was positive in March–May. We believe that abundant rainfall, like higher temperature, promoted its flowering while the higher precipitation in March–May prolonged the duration of flowering. Therefore, in 2013, when precipitation was anomalously low in February and this was followed by a sudden surge in rainfall in March–June, the PFD of *B*. *scandens* was significantly delayed.

The majority of the 12 species analyzed here had a significant response to monthly precipitation and mean monthly temperature within the 0–2 preceding months of their peak flowering date. This was consistent with the conclusions of many previous studies^3,7,8,46^ reporting that hydrothermal conditions of that stage affected the physiological activities of plants and determined the timing of flowering. However, we found the mean temperature and precipitation in the previous November and previous December were also correlated with flowering in some species. This may occur because the climate of autumn and winter affected senescence and budding, which had a strong temporal relationship with flowering, especially for tropical deciduous plants^47^. Lambert *et al*. found that greater amounts of summer precipitation in the previous year led to earlier flowering in *Erythronium grandiflorum* at the Rocky Mountain Biological Laboratory^48^. Moreover, studies in Hainan Island, Hainan Province^49^, and Guiyang, Guizhou Province^50^, China, came to similar conclusions in support of the hypothesis that the effects of precipitation on phenology had a hysteretic nature.

In the present study, more species exhibited a stronger response to monthly precipitation than to temperature. In tropical or subtropical regions^50^, such as some tropical Asian forests^11,51^, the variability of precipitation was greater than that of temperature, so rainfall became an important factor for inducing flowering. In the central Amazon Basin, most flowering was concentrated during the transition from the dry to the rainy season and this provided advantages related to insect pollination and seed dispersal^52^. Four out of 12 species in our study (*C*. *burmanniii*, *M*. *paniculatus*, *L*. *gracile*, and *L*. *monopetala*) were wind-pollinated while the others were insect-pollinated. We did not find explicit differences between these two types in the timing of flowering or the response to MP and MMT, presumably because this study analyzed only a very limited number of species.

In addition, some previous studies have shown that a relationship exists between FFD and the SD of first flowering day, and implied a greater amount of variation occurs in early-flowering species than those flowering later^7,42^, but we did not find significant correlation between the SD of PFD and PFD itself (P value = 0.743). Then we defined early-flowering species as those species with average PFDs that occurred before June. These species included *C*. *burmannii*, *S*. *jambos*, *L*. *monopetala*, *B*. *scandens*, *S*. *lanceolata*, *T*. *cochinchinensis*, *P*. *asiatica*; the remaining species were defined as late-flowering species. The PFDs of four out of five late-flowering species were significantly correlated with MP in phase-2; the flowering dates of two species advanced significantly in 2013. In contrast, the PFD of only one out of seven early-flowering species (*C. burmannii*) was significantly correlated with MP in phase-2. Then, we found no significant difference of interannual SDs between early- (11.09 days) and late-flowering (18.32 days) species (P value = 0.158). However, the difference of interannual SDs could also be caused by different observation frequencies of observation (twice a month in spring and summer; once a month in autumn and winter). In the future, we are trying to make it twice a month all year long.

According to the 30-year climate data of 1981–2010, the amount of rainfall received after April each year of the rainy season has increased and the MPs of the months of May–September were all above 200 mm, while it remained below 100 mm in other months. The MP of the rainy season, which varied more sharply than at other times, mainly affected the late-flowering species, so the PFDs of late-flowering species were more variable accordingly. Körner believed that, in extratropical areas, photoperiod is rather invariable when compared with temperature in spring; therefore, tracking temperature would be a more risky life strategy for early successional, shorter-lived species, in order to profit from climate warming and thus gain a competitive advantage over photoperiod-sensitive species^4^. According to this view, was a similar strategy adopted by late-flowering species that track MP in our study? If so, the early-flowering species would be mainly composed of late successional species. A number of these are dominant in this community. We found that the three most abundant species, *P*. *asiatica*, *S*. *lanceolata*, and *C*. *burmannii*, were all early-flowering and their PFDs lacked a significant correlation with MP or MMT. However, we cannot draw some conclusions until we have more data. This is an interesting question to be explored in future work.

In conclusion, we found that: (1) The peak flowering dates of 12 species in Guia Hill were concentrated in spring and summer. (2) The majority of these 12 species had a significant response to monthly precipitation and monthly mean temperature during 0–2 months before their 5-year mean PFDs. (3) The mean temperature and precipitation in the previous November and previous December were also factors affecting the timing of flowering. (4) Eleven out of 12 species had varying degrees of response to abnormal climate in 2013 or 2016. These results were consistent with their response to temperature and precipitation. (5) Precipitation in their optimum period had a more effective impact on the PFDs of 12 species than temperature. (6) Late-flowering species were more responsive to precipitation in 0–2 months before than early-flowering species.

## Electronic supplementary material

dataset 1

## Data Availability

All data generated or analyzed during this study are included in this published article (and its Supplementary Information files).
