# Supplementary material for: Rapid Shifts of Peak Flowering Phenology in 12 Species under the Effects of Extreme Climate Events in Macao
Source: Sci Rep. 2018 Sep 17;8:13950. doi: 10.1038/s41598-018-32209-4 (PMC6141562; doi:10.1038/s41598-018-32209-4)
Supplement: Supplementary file 1 — dataset 1 [file 41598_2018_32209_MOESM1_ESM.pdf]

Rapid Shifts of Peak Flowering Phenology in 12 Species under the Effects of Extreme Climate Events in Macao

Jianhao Zhang[1,2], Qifei Yi[1*], Fuwu Xing[1], Chunyan Tang[1], Lin Wang[1], Wen Ye[1], Ian Ian Ng[3], Tou I Chan[3], Hongfeng Chen[1] & Dongming Liu[1]

[1]South China Botanical Garden, The Chinese Academy of Sciences, Guangzhou 510650, China. [2]University of Chinese Academy of Sciences, Beijing 100049, China. [3]Department of garden and Green Areas, Civil and Municipal Affairs Bureau of Macao Special Administrative Region, Macao 999078, China.

Correspondence and requests for materials should be addressed to Q.Y. (email: yiqifei@scbg.ac.cn)

PFDs of individuals of *Sterculia lanceolata*

2012	2013	2014	2015	2016
109.5	68	92	91	107
109.5	79	92	97	107
115	81	99	97	107
116	81	109	97	119
116	81	113	97	119
119	81	113	97	119
120	81	113	97	119
120	83	113	99	119
120	91	113	99	119
120	100	116	105	119
120	105	116	105	119
120	105	116	105	119
126	107	123	110.5	119
126	113	123	111	119
126.5	113	123	111	121
127	113	123	111	126
127	113	123	111	126
127	113	123	111	131
127	113	123	111	131
127	114	123	111	131
127	114	123	113	131
127	114	123	119	131
127	121	123	119	138
127	121	123	119	138
127	121	123	119	138
127	126	126	119	138
133	126	126	119	138
133	126	126	119	138
133	129	130	119	138
133	132	130	119	138
133	132	130	119	138
133.5	132	130	119	145
133.5	132	130	119	145
133.5	132	134	122	145
133.5	132	134	125	145
133.5	139.5	141	125	162
135.5	139.5	150	127	162

141	140	168	133	
141	140	243	133	
143	145	243	133	
144	145	260	133	
153.5	151	284	136	
158	151		139	
215	207		139	
229	234		139	
286			139	
			150	
			158	
			161	
			161	
			209	

PFDs of individuals of *Mallotus paniculatus*

2012	2013	2014	2015	2016
192	220	189	230	212.5
213	220	223	230	230
280	220	226	230	230
280	233.5	226	230	230
	234	226	230	230
	234	226	230	230
	261	226	245	230
		226	245	230
		226	245	230
		235.5	245	230
		235.5	245	230
		235.5	245	230
		243	245	230
		243	245	230
		243	245	230
		252.5	245	230
		252.5	245	230
		263	245	230
		263	245	286
		263	245	
		272.5	245	
		282	245	
		282	245	
		282	258.5	
			260	
			287	

PFDs of individuals of *Lophatherum gracile*

2012	2013	2014	2015	2016
248.5	138.5	263	260	246.5
263	187	263	287	264
263	247.5	263	287	255.3
263	261	263		
263	261	263		
263	261	263		
263	261	263		
263	261	263		
263	261	263		
263	261	272.5		
		282		
		322		

PFDs of individuals of *Breynia fruticosa*

2012	2013	2014	2015	2016
126.5	132	190.5	110.5	121
248.5	79	139.5	304	121
187	295.5		207	121
	90.5			

PFDs of individuals of *Litsea monopetala*

2012	2013	2014	2015	2016
108	106.5	91.5	127	146
108	106.5	91.5	127	146
108	114	101	119	146
115	113		230	

PFDs of individuals of *Desmos chinensis*

2012	2013	2014	2015	2016
166	100	168	161	184
171.5	113	168	161	184
229	147	168	174.5	186
263	151	168	209	217
	151		230	
	151			
	178.5			
	178.5			
	178.5			

PFDs of individuals of *Psychotria asiatica*

2012	2013	2014	2015	2016
112	45	66.5	66.5	88
127	55.5	112	99	119
133	56.5	113	101.5	119
133.5	56.5	113	105	119
133.5	56.5	115.5	112	121
133.5	60.5	122.5	115	121
133.5	68	122.5	122.5	126
133.5	79.5	123	122.5	126
133.5	79.5	130	123	126
133.5	79.5	130	130	126
133.5	81	130	130	126
133.5	90.5	130	130	126
133.5	92.5	130	130	126
133.5	92.5	130	130	126
133.5	104.5	130	130	126
133.5	108	130	130	126
133.5	114	130	130	126
133.5	114	130	130	126
133.5	114	130	130	126
133.5	114	130	130	126
133.5	116	130	130	126
133.5	120.5	130	130	126
133.5	120.5	130	130	126
133.5	120.5	130	130	126
133.5	120.5	130	130	126
133.5	121.5	132	130	126
133.5	125.5	139	130	126
134	125.5	140.5	132	126
134	125.5	140.5	136.5	126
134	125.5	140.5	140.5	131
134	125.5	140.5	140.5	131
134	125.5	140.5	140.5	131
134	125.5	140.5	140.5	131
134	125.5	140.5	140.5	131
134	125.5	140.5	140.5	138

134	125.5	140.5	140.5	138
134	125.5	140.5	140.5	138
134	129	140.5	140.5	138
134	129	140.5	140.5	138
134	129	140.5	140.5	138
134	129	140.5	140.5	138
134	131	140.5	140.5	138
134	132	140.5	140.5	138
134	132	140.5	140.5	138
134	132	140.5	140.5	138
134	132	140.5	140.5	138
134	132	140.5	140.5	138
134	132	140.5	140.5	138
134	132	140.5	140.5	138
135	132	144	140.5	138
135	132	144	144	138
142	132	144	144	138
142	132	144	144	138
142	132	147	144	138
142	132	147	144	138
142	132	147	147	138
142	132	147	147	138
142	132	150	147	138
142	132	150	147	138
143	132	150	147	138
144	138.5	150	147	138
144	138.5	150	147	138
144	138.5	150	147	138
144	138.5	150	149	138
144	138.5	150	149	138
144	138.5	150	150	138
144	138.5	150	150	138
144	138.5	151	150	138
144	139.5	151	150	143.5
147	139.5	151	150	145
147	139.5	151	150	145
147	139.5	151	151	145
151	139.5	151	151	145
151	139.5	151	151	145

151	139.5	151	151	145
151	139.5	151	151	145
151	143.5	151	151	145
151	145	151	151	145
151	145	151	151	145
151	145	151	151	145
151	145	151	151	145
151	145	151	151	145
151	145	151	151	145
151	145	151	151	145
151	145	151	151	145
151	145	151	151	145
151	145	151	151	151
153	145	151	151	151
153	145	151	151	151
153.5	145	151	151	151
154	145	151	151	151
154	145	151	151	151
154	145	151	151	151
154	151	151	151	151
154	152.2	157.5	151	151
154	152.5	157.5	151.5	151
154	152.5	157.5	157	151
154	152.5	157.5	157	155.5
154	152.5	161	157	155.5
154	152.5	161	157	155.5
154	152.5	161	157	155.5
154	152.5	161	157	162.5
154	152.5	161	157	163
154	153	161	157	163
154	153	161	157	163
154	159.5	161	157	163
154	159.5	161	157.5	163
154	159.5	161	157.5	163
154	159.5	161	157.5	163
154	159.5	161	157.5	163
154	159.5	161	157.5	163
154	159.5	161	157.5	163
154	159.5	161	157.5	163

154	159.5	161	158	163
154	162	161	158	163
154	164	168	161	163
154	164	168	161	163
154	164	168	161	163
154	164	168	163	163
154	164	168	163	163
154	164	168	164	168.5
154	164	168	164	168.5
154	164	168	168	170
158	167	178	168	170
158	167	178	168	187.5
158	167	178	168	187.5
158	167	178	170	187.5
158	167	178	170	195
158	167	178	170	195
158	167	179	171	212.5
158	167	179	171	212.5
160	167	179	171	
164	167	179	171	
164	177	179	171	
164	178.5	179	171	
164	178.5	185	171	
164	178.5	185	171	
164	178.5	185	174	
164	181	185	175	
164	191.5	185	178	
164	191.5	185	178	
164		185	178	
164		188	178	
164		189	179	
164		189	179	
171		196	179	
171		198	185	
171		206	185	
174.5		207	185	
179		207	186	
185		207	186	
185		207	188	

185		207	188	
185		218.5	189	
192		228	189	
192		247	193	
192			193	
192			194	
192			196	
199.5			206	
203.5			213	
214				

PFDs of individuals of *Benkara scandens*

2012	2013	2014	2015	2016
101	114	115.5	104.5	95.5
108	114	115.5	104.5	107.5
112.5	114	123	104.5	114.5
116	116	123	110.5	114.5
118.5	125.5	130	110.5	121
119.5	128	132	111	131
120	138.5	132	113	138
125.5	151	132	113	138
	152.5		119	
	177		119	
			125	

PFDs of individuals of *Syzygium jambos*

2012	2013	2014	2015	2016
72.5	41.5	91.5	90.5	60
146	82.5	106	90.5	60
87.5	89	113	96.5	72.5
	106.5		96.5	97
	106.5		96.5	131.5
	106.5		96.5	93.5
	106.5		96.5	
	114		96.5	
			104.5	
			104.5	
			105	
			113	

PFDs of individuals of *Syzygium levinei*

2012	2013	2014	2015	2016
212	234	206	224	230
212	261	206	209	230
212		206	237.5	230
		226		

PFDs of individuals of *Triadica cochinchinensis*

2012	2013	2014	2015	2016
101	116	115.5	105	107
171	125.5	147	133	163
171	139.5	157.5	133	163
94	139.5	157.5	133	195
132.5			149.5	
151				
151				

PFDs of individuals of *Cinnamomum burmannii*

2012	2013	2014	2015	2016
87	55.5	70	70	60
87	65	70	82	70.5
88	65	77	82	70.5
88	65	77	90.5	76
88.5	71.5	77	90.5	76
88.5	73	77.5	90.5	87.5
88.5	73	84	90.5	87.5
88.5	73	84	96.5	87.5
88.5	73	84	96.5	87.5
88.5	73	84	96.5	90.5
88.5	73	84	96.5	90.5
94	73	84	96.5	90.5
94	73	84.5	99	90.5
94	73	84.5	99	90.5
94	73	84.5	104.5	95.5
94	73	84.5	104.5	95.5
94	81	84.5	104.5	95.5
94	81	91.5	104.5	95.5
94	89	91.5	104.5	97
95.5		91.5	104.5	97
95.5		91.5	105	99.5
95.5		91.5	105	102
95.5		91.5	105	102
95.5		91.5	110.5	102
102		91.5	110.5	102
103		91.5	110.5	102
109.5		91.5	110.5	114.5
		91.5	110.5	114.5
		94	110.5	139.5
		94	110.5	145
		98.5	125	230
		99	135	
		99		
		99		
		99		
		99		

		108		
		108.5		
		136.5		
		103.5		

Table Monthly Mean Temperature (°C) for 2012–2016.

Year	Jan.	Feb.	Mar.	Apr.	May	Jun.	Jul.	Aug.	Sep.	Oct.	Nov.	Dec.	Mean
2012	13.64	14.95	18.4	23.26	27.17	27.9	28.44	29.23	27.93	25.65	21.45	16.95	22.91
2013	15.74	18.5	20.13	21.26	25.66	28.18	28.16	28.58	27.51	25.56	21.46	15.29	23.00
2014	15.94	14.63	18.45	22.65	26.44	29.27	29.9	29.05	29.06	26.19	22.07	15.27	23.24
2015	15.8	16.82	19.35	22.71	27	29.23	28.92	29	28.15	25.92	23.96	17.21	23.67
2016	15.2	14.34	17.04	22.91	26.39	28.92	29.48	27.93	27.17	26.66	21.39	18.94	23.03

Table Monthly precipitation (mm) for 2012–2016.

Year	Jan.	Feb.	Mar.	Apr.	May	Jun.	Jul.	Aug.	Sep.	Oct.	Nov.	Dec.	Annual total
2012	42.16	27.45	19.74	316.3	123.79	201	451.57	110.57	57	62	90	83.6	1585.18
2013	1.03	3.55	138.57	208.7	488.35	283	363.73	229.4	472.33	9	56.4	44.33	2298.39
2014	0	27	85.27	99.33	572.87	171	197.87	247.33	86.73	82.4	48.3	55.8	1673.9
2015	28.52	41.72	12.61	46.9	310.31	142.6	117.8	147.87	147.3	243	117	6.8	1362.43
2016	250.13	31	194.07	161.8	204.33	273.73	177.47	389.8	121.93	110	35.8	11.2	1961.26

Table Significant correlations between eight species and monthly precipitation (MP) or mean monthly temperature (MMT) in the optimum period.

Species	MMT		MP	
	Period-1	Period-2	Period-1	Period-2
Syzygium jambos	negative **		negative	negative
Mallotus paniculatus	negative **		negative	negative **
Breynia fruticosa		positive **	negative	negative **
Desmos chinensis		positive	negative	negative
Lophatherum gracile				negative
Triadica cochinchinensis		negative		
Benkara scandens				negative, positive **
Cinnamomum burmannii				positive **

** correlation is significant at the 0.01 level.
